# Case Report of Atlantoaxial Rotatory Fixation after Cochlear Implantation

**DOI:** 10.1155/2016/6486271

**Published:** 2016-06-02

**Authors:** Takahiro Nakashima, Keiji Matsuda, Takumi Okuda, Tetsuya Tono, Minoru Takaki, Tamon Hayashi, Yutaka Hanamure

**Affiliations:** ^1^Department of Otorhinolaryngology, Head and Neck Surgery, Faculty of Medicine, University of Miyazaki, 5200 Kihara, Kiyotake-cho, Miyazaki 889-1692, Japan; ^2^Department of Otorhinolaryngology, Head and Neck Surgery, Kagoshima City Hospital, 37-1 Kamiaratacho, Kagoshima 890-8760, Japan

## Abstract

Atlantoaxial rotatory fixation (AARF) is a relatively rare condition and is mainly seen in children. We report of a 7-year-old girl suffering from AARF after cochlear implantation (CI). Fortunately, early diagnosis based on three-dimensional computed tomography (3DCT) was made, and the patient was cured with conservative therapy. Nontraumatic AARF, which is also known as Grisel's syndrome and occurs subsequent to neck infections or ear, nose, and throat (ENT) surgery, represents only a small fraction of AARF cases. Two factors are mainly thought to contribute to the pathogenesis of the condition estimated, namely, (i) neck immaturity in children and (ii) infiltration by inflammatory mediators around the upper neck joint, easily permitted by the neck vasculature. AARF should be suspected in case of torticollis developing after ENT surgery.

## 1. Introduction

Atlantoaxial rotatory fixation (AARF) is one of the pathological conditions potentially leading to torticollis. Although many cases are reported as a consequence of trauma, AARF can also develop subsequent to infectious diseases in the neck and ear, nose, and throat (ENT) surgery. We identified a case of AARF after cochlear implantation (CI) representing a rare but very important case for otolaryngologists, because it was a postoperative complication. The purpose of this report is to remind otolaryngologists of the fact that AARF may occur as an early complication to ear surgery.

## 2. Case Presentation

A 7-year-old female presented with bilateral profound sensorineural hearing loss. She had a history of low birth weight (2190 grams), operation for congenital diaphragmatic hernia, and no automatic auditory brainstem response while in the neonatal intensive care unit.

After being discharged from hospital, thorough examinations were performed by otolaryngologists. Physical examinations revealed normal auricles, external auditory canals, and tympanic membranes. Her neck motion was normal and no systemic malformations were detected. Audiologic tests including auditory brainstem response, auditory steady state response, and conditioned orientation reflex audiometry with and without hearing aid revealed bilateral profound sensorineural hearing loss. Computed tomography (CT) and magnetic resonance imaging of the temporal bones showed no inner or middle ear malformation. The patient did not have Down syndrome and had no apparent mental and growth retardation. Given the diagnosis of congenital deafness, hearing aids were provided to the patient when she was 6 months old. The patient was reevaluated 10 months after; however, any efficacy of the hearing aids could not be demonstrated.

We explained to the family that CI was recommended before the patient reached the age of 2 years; however, approval of the operation could not be obtained. CI in the right ear was eventually performed when the patient was 7 years old. There were no complications, and the intraoperative change in neck position was minimized. The length of anesthesia and operation was 255 min and 170 min, respectively. After surgery, the patient had no neurological deficits.

The day after surgery, the patient complained of dull neck pain in her right side. Physical examination showed no mass but weak, bilateral neck tenderness. Neck movement was restricted and her head was tilted to the right side, rotating to the other. Conservative therapy with oral acetaminophens was applied, but clinical features remained.

**Figure 1:**
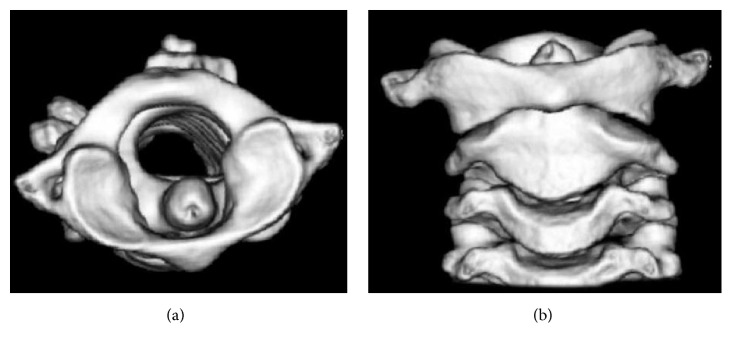
Axial (a) and coronal (b) view of 3DCT before treatment. The atlas was rotated without dislocation of the odontoid process in axial view. The axis showed slight oblique position against atlas in coronal view.

**Figure 2:**
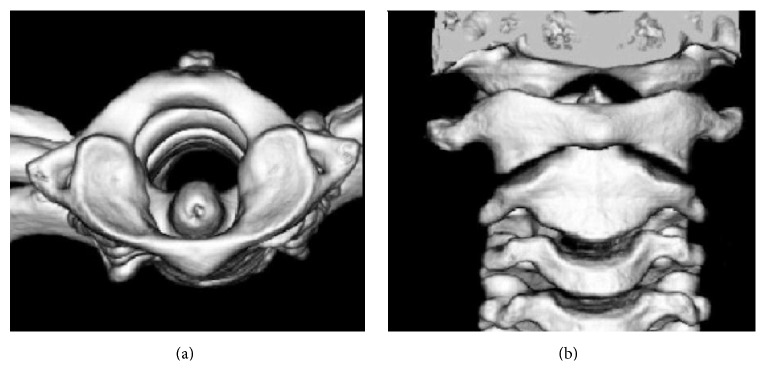
Axial (a) and coronal (b) view of 3DCT after treatment revealed normal appearance in atlantoaxial joint.

On the 5th day after surgery, the patient consulted an orthopedic specialist. Three-dimensional CT (3DCT) revealed asymmetry in atlantoaxial joint and rotation of the atlas with the odontoid process acting as pivot (Figure 1). These findings lead to the diagnosis of AARF, and the patient was treated with indirect cervical traction at bedside for 1 week, and she was eventually discharged with a soft cervical collar for another week. Follow-up 3DCT showed normal appearance of the atlantoaxial joint (Figure 2). Her neck moves normally with no pain.

## 3. Discussion

Atlantoaxial rotatory fixation is a relatively rare condition that may result in torticollis. Atlantoaxial rotatory dislocation or atlantoaxial rotatory subluxation also leads to torticollis. These terms have often been used interchangeably; however, they are pathophysiologically different conditions. AARF has sometimes been described as fixed atlantoaxial rotatory dislocation [1] or atlantoaxial rotatory fixation-subluxation [2]. It may be a result of trauma, inflammation because of upper respiratory tract infection, ENT surgery, or unknown factors, with trauma being the most common eliciting factor. AARF due to nontraumatic causes, such as upper respiratory tract inflammation or ENT surgery, is relatively rare. The former includes cervical lymphadenitis [3] and retropharyngeal abscess [4]. The latter includes adenotonsillectomy [5], tympanoplasty [6], and otoplasty [7]. Nontraumatic AARF, usually known as Grisel's syndrome, is diagnosed on the basis of an algorithm including a history of ENT surgery or ENT infection, development of torticollis a few days after onset of operation/infection, rotation and slight flexion of the head, painful active or passive rotation of the head, elevated inflammatory parameters eventually normalized with no fever, and atlantoaxial subluxation and rotation on CT scan [8]. Weißkopf et al. [1] reported 26 cases in which two cases were due to pediatric ENT surgery. Karkos et al. [9] summarized 96 cases of Grisel's syndrome in which the main causes were infection (48%) and postadenotonsillectomy (31%); tympanomastoidectomy accounted for merely 1% of the cases.

The higher incidence of AARF in children may be ascribed to anatomical characteristics, including physiologically immature neck musculature, a rotation angle greater than adults because of a larger atlas-dens interval, and ligamentous laxity. In addition, pharyngovertebral veins draining the lymphatics of the retropharyngeal space facilitate infiltration by inflammatory mediators around the atlantoaxial (AA) joint leading to inflammatory changes around the AA joint. Battiata and Pazos [10] proposed a two-hit hypothesis regarding the pathogenesis of Grisel's syndrome. According to this model, cervical ligamentous laxity seen in pediatric populations at baseline is the first hit. Spasm of cervical muscles induced by inflammatory mediators is the second hit. Subsequently, subluxation of the AA joint occurs.

During ENT surgery, the neck of the present case may have been hyperflexed and excessively rotated. This situation may have affected the AA joint to an extent that allowed for AARF to develop. The history of low birth weight, operation for congenital diaphragmatic hernia, and the long term tracheal intubation to manage and maintain respiratory functions in her infanthood might have led to the condition called AA instability, resulting from immature neck musculature and ligament looseness.

According to plain tomography, Fielding and Hawkins [11] divided AARF into four categories. Roche et al. [12] pointed out that type 1 in the Fielding classification might erroneously be classified as type 2 in children because the atlas-dens interval of the child is larger than that of the adult. In addition, Roche et al. emphasized the usefulness of dynamic studies including CT as well as 3DCT imaging.

Treatment for AARF categorized as type 1 according to the Fielding classification and defined as rotary fixation with no anterior displacement, with the odontoid acting as the pivot point, is generally conservative. Most of these cases are cured without any complications. However, lately diagnosed or inappropriately treated cases sometimes require surgical intervention. Early diagnosis and consulting orthopedicians are therefore important.

Concerning AARF developing after CI operation, no reports written in English have been found in search on Medline. The age of patients receiving CI operations appears to decrease and the number of CI operations before the age of 2 years is increasing. Such children sometimes exhibit multiple anomalies or growth retardation, which means that they have a high risk of developing AARF.

It is crucial to try and prevent postoperative AARF. Both anesthesiologist and surgeon must check the mobility of the patient's neck prior to operation. Kim et al. [13] proposed a set of guidelines for prevention of AARF. These guidelines consist of the following elements: (1) informed consent, (2) preoperative check, (3) appropriate surgical position, and (4) postoperative check. Regarding the surgical position, appropriate use of rotation on the operation table is valuable. Because the range of cervical rotation is limited [12], excessive rotation of the neck should be avoided. In addition, there is a need to be aware of high-risk patients, including patients with AA instability typically seen in small children and children with developmental abnormalities, such as Down syndrome.

## 4. Conclusion

AARF after ear surgery is a rare condition. However, ENT surgery is one of the risk factors for developing nontraumatic AARF. Otolaryngologists should keep in mind that this disease may occur as an early complication of ear surgery, especially in small children.
